# Health-related quality of life of patients with resistant/intolerant chronic phase chronic myeloid leukemia treated with asciminib or bosutinib in the phase 3 ASCEMBL trial

**DOI:** 10.1038/s41375-023-01888-y

**Published:** 2023-04-14

**Authors:** Delphine Réa, Carla Boquimpani, Michael J. Mauro, Yosuke Minami, Alex Allepuz, Vikalp Kumar Maheshwari, Denise D’Alessio, Ying Wu, Rachael Lawrance, Sarunas Narbutas, Giora Sharf, Andreas Hochhaus

**Affiliations:** 1 Hôpital Saint-Louis, CIC 1427 Paris, France; 2 HEMORIO, State Institute of Hematology Arthur de Siquiera Cavalcanti, Rio de Janeiro, Brazil; 3 Oncoclínica Centro de Tratamento Oncológico, Rio de Janeiro, RJ Brazil; 4 Memorial Sloan-Kettering Cancer Center, New York, NY USA; 5 National Cancer Center Hospital East, Kashiwa, Japan; 6 Novartis Pharma AG, Basel, Switzerland; 7 Novartis Healthcare Pvt. Ltd., Hyderabad, India; 8 Novartis Services Inc, East Hanover, NJ USA; 9 Adelphi Values, Bollington, Cheshire UK; 10 Youth Cancer Europe and CML Advocates Network, Vilnius, Lithuania; 11 Leukemia Patient Advocates Foundation, Netanya, Israel; 12 Universitätsklinikum Jena, Jena, Germany

**Keywords:** Drug development, Cancer therapy

## Abstract

In ASCEMBL, an open-label, randomized Phase 3 study, asciminib demonstrated superior efficacy and better safety profile compared with bosutinib in patients with chronic myeloid leukemia in chronic phase (CML-CP) previously treated with ≥2 tyrosine kinase inhibitors. Health-related quality of life (HRQOL) reported by patients is key to understanding the benefit and impact of treatment on patients’ lives, and is becoming increasingly important as the life expectancy of CML-CP patients increases and patients require long-term treatment. In ASCEMBL, patients completed questionnaires to assess CML symptoms and interference with daily life (M.D. Anderson Symptom Inventory – CML [MDASI-CML]), general HRQOL (five-level EQ-5D [EQ-5D-5L], Patient Global Impression of Change – CML [PGIC-CML]), and impact of CML on working life and activity (Work Productivity and Activity Impairment questionnaire – CML [WPAI-CML]). Patients’ CML symptoms and HRQOL remained stable during 48 weeks of treatment with asciminib, with a general trend for decreased CML symptom severity, particularly for fatigue, and improvement in HRQOL. A clinically meaningful increase in diarrhea severity was observed in patients treated with bosutinib compared to asciminib. These data provide better understanding of the patient perspective and treatment impact on HRQOL in a later-line setting, where little information has been published to date.

## Introduction

Chronic myeloid leukemia (CML) accounts for ~15% of all new cases of leukemia [1]. In 2001, the first oral targeted therapy authorized for CML was imatinib, an adenosine triphosphate (ATP)-competitive tyrosine kinase inhibitor (TKI) therapy that selectively blocks the activity of ABL kinases [2]. The success of imatinib and second- and third-generation TKIs has dramatically changed the outcome for CML patients. However, patients with CML require potentially life-long treatment with TKIs, increasing the risk of toxicities associated with off-target effects. For patients in the third-line setting and beyond, the choice and sequencing of TKIs remains individual, with the complexity of safety and tolerability considerations in the context of intolerance or resistance to multiple prior treatments [3–6].

Each TKI has a specific safety profile with associated side effects that can ultimately affect adherence to therapy and health-related quality of life (HRQOL). Hence, patient-reported outcomes (PROs) are critical in precisely reporting and evaluating patients’ HRQOL, symptoms, and side effects and the drug tolerability of long-lasting, daily treatments [7]. There are limited data regarding HRQOL in later-line CML patients [8]; therefore, it is important to make such data available to patients and physicians to inform treatment decisions.

Asciminib, unlike existing ATP-competitive TKIs, allosterically inhibits BCR::ABL1 through Specifically Targeting the ABL Myristoyl Pocket (STAMP) [9, 10]. Pre-clinical data showed that, because asciminib is specific for ABL kinases (ABL1, ABL2, and the chimeric BCR::ABL1), it does not elicit off-target kinase-mediated effects seen with approved ATP-competitive TKIs [9]. The pre-clinical findings translated in the clinical setting of the ASCEMBL study in an improved safety profile and tolerability of asciminib as compared with the second-generation TKI bosutinib [11]. Approval of asciminib was first granted by the United States Food and Drug Administration (FDA) in 2021 for the treatment of adults with Philadelphia-positive CML in chronic phase (CP) previously treated with ≥2 TKIs and for those with the BCR::ABL1 T315I mutation [12].

ASCEMBL (NCT03106779) is the first randomized controlled study comparing treatments for patients with CML-CP who were resistant or intolerant to at least two prior TKIs. Asciminib (40 mg BID) demonstrated superior efficacy compared with bosutinib (500 mg QD) to meet the primary study endpoint of major molecular response (MMR) at 24 weeks. MMR rate at week 24 was 25.5% on asciminib versus 13.2% on bosutinib; the absolute difference in MMR rates after adjusting for major cytogenetic response status at baseline was 12.2% (95% confidence interval [CI], 2.19%–22.30%) [refs. [11, 13]]. The difference in MMR between treatments was maintained at week 48, with an MMR rate of 29.3% with asciminib versus 13.2% with bosutinib [14]. In both the primary analysis results (week 24) and the interim week 48 analysis, after a median duration of exposure of 43.4 weeks and 67.1 weeks on asciminib, and 29.2 weeks and 29.7 weeks on bosutinib, respectively, fewer patients treated with asciminib experienced adverse events that led to treatment discontinuation compared to bosutinib (5.8% versus 21.1% and 7.1% versus 25.0%, respectively) [11, 14]. Median dose intensity after 48 weeks was 79.8 (range, 33–80) and 463.8 (range, 181–566) mg/day for asciminib and bosutinib, respectively.

Previously presented HRQOL data based on ASCEMBL 24-week analysis indicated that patients treated with asciminib showed improvement in treatment-related symptoms and HRQOL compared to baseline and relative to bosutinib [15]. Here we report the long-term impact on CML-specific symptoms, HRQOL, and work productivity in patients with CML-CP treated with asciminib compared to those treated with bosutinib up to 48 weeks of treatment. The findings presented here are an extension of the results presented at SOHO 2021 [15], which was the first time PRO data have been published for patients with CML treated with asciminib.

## Materials and methods

### Study design

**Fig. 1:**
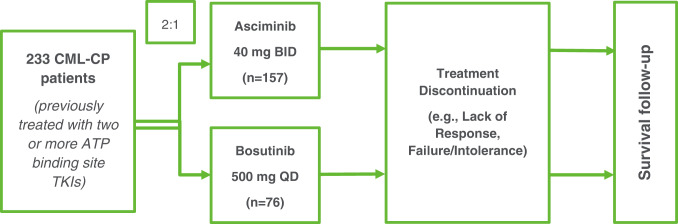
Schematic of Study Design. ATP: adenosine triphosphate, BID: twice daily, CML-CP: chronic myeloid leukemia in chronic phase, QD: once daily, TKI: tyrosine kinase inhibitor Switch to asciminib allowed for bosutinib-treated patients meeting the lack of efficacy criteria based on the 2013 European LeukemiaNet recommendations for second-line TKI therapy [29]; however, PRO assessments were not collected after treatment switch.

ASCEMBL is a phase 3, multi-center, open-label, randomized clinical trial (Fig. 1 [ref. [11]]). The protocol was approved by the sites’ institutional review boards and all patients provided written informed consent. A total of 157 patients were randomized to asciminib 40 mg twice daily, and 76 patients were randomized to bosutinib 500 mg once daily. Patients remained on randomized treatment for at least 96 weeks unless discontinued from treatment due to lack of efficacy or disease progression, unacceptable toxicity and/or at the discretion of the investigator or the patient. Further details on the study design have been presented previously [11].

#### Frequency of PRO assessments

Four self-reported PRO questionnaires (M.D. Anderson Symptom Inventory – CML [MDASI-CML], five-level EQ-5D [EQ-5D-5L], Patient Global Impression of Change – CML [PGIC-CML], and Work Productivity and Activity Impairment – CML [WPAI-CML]), each assessing different aspects of patients’ HRQOL and experience on treatment, were administered electronically at baseline (except PGIC-CML) as well as during clinic visits at weeks 4, 8, 12, 16, 24, 36, 48, and 96 while patients remained on randomized treatment. PRO questionnaires were translated into 35 languages; patients completed them in the most familiar language. The PRO analyses presented here are based on the 48-week study data cut-off.

### PRO questionnaires

#### MDASI-CML: CML symptoms and interference with daily life

The MDASI-CML is a validated 26-item, multi-symptom PRO questionnaire for clinical and research use. The MDASI-CML includes 13 core symptoms found to have the highest frequency and/or severity in patients with various cancers and treatment types (assessing pain, fatigue, nausea, disturbed sleep, feeling distressed, shortness of breath, problems remembering things, lack of appetite, feeling drowsy, dry mouth, feeling sad, vomiting, numbness or tingling) and 7 symptoms specific to CML (diarrhea, swelling, rash/skin change, muscle soreness/cramping, bruising/bleeding easily, malaise, and headache) [16–18].

The MDASI-CML assesses the severity of symptoms at their worst in the last 24 h on a 0–10 scale, with 0 being “not present” and 10 being “as bad as you can imagine.” The symptom severity score is the mean of the 13 core symptom items and 7 CML-specific symptom items, representing overall symptom severity.

The MDASI-CML also measures how much symptoms have interfered with six daily activities: general activity, mood, work, relations with others, walking, and enjoyment of life. Interference is rated on a 0–10 scale, with 0 being “did not interfere” and 10 being “interfered completely.” The symptom distress score is the mean of the six interference items, representing overall symptom distress.

#### EQ-5D-5L: patients’ general HRQOL

The EQ-5D-5L is a standardized measure of health utility providing a single index value for one’s health status and is used for economic evaluations of healthcare. The EQ-5D-5L is composed of a descriptive system of five dimensions and a visual analogue scale (VAS). The VAS component is a measure of self-rated health, rated on a scale from 0 (worst imaginable health state) to 100 (best imaginable health state) [19].

#### PGIC-CML: patients’ impression of change in CML symptoms

The PGIC-CML is a single-item scale that asks: “Since the start of the treatment you’ve received in this study, your Chronic Myeloid Leukemia (CML) symptoms are…” with seven response options: (1) very much improved, (2) much improved, (3) minimally improved, (4) no change, (5) minimally worse, (6) much worse, and (7) very much worse. For the analysis reported herein, responses were grouped into “improved” (1–3), “no change” (4) and “worsened” (5–7). The PGIC is a commonly used measure of improvement or deterioration over time relative to treatment.

#### WPAI-CML: impact on work and activity

The WPAI-CML is a six-item PRO questionnaire that assesses the effect of CML on patients’ ability to work and perform regular activities (recalling the previous 7 days). In patients that report that they are working, three scales are calculated: work time missed (absenteeism), impairment while working (presenteeism), and overall work impairment (productivity). In addition, the questionnaire asks all patients (regardless of if they are working or not) about their general activity impairment. Scores are expressed as percentages out of 100, with higher scores indicating greater impairment [20].

#### Interpretation of PRO scores—Meaningful changes

For consistency across the PRO questionnaires, and due to the lack of published data relating to minimally important difference (MID) thresholds for the CML-specific instruments (MDASI-CML and WPAI-CML), a threshold of 1.5 points or 15% is used in this analysis as a guide to clinically meaningful thresholds, in line with interpretation guidelines from certain health technology assessment (HTA) agencies [21]. For the EQ-5D-5L VAS, a published MID of 7 points is commonly reported in the literature [22]. The PGIC-CML is directly expressed in levels of changes (improved, no change, worsening), more clearly aligning with interpretation of meaningful changes.

### Statistical analysis methods

The completion rate of each PRO questionnaire was summarized as a percentage out of the number of randomized patients who were expected at that visit; patients expected at each post-baseline visit were those who remained on randomized treatment and had not progressed, switched treatment, died, or withdrawn from the study for any other reasons at that visit.

The MDASI-CML, EQ-5D-5L VAS, and WPAI-CML were summarized at baseline and at each subsequent visit descriptively using means and standard deviation. The PGIC-CML was summarized as proportions of patients who reported improvement, no change, or worsening at each visit.

To compare the difference between treatment arms in changes from baseline over time in PRO scores up to week 48 (longitudinal analysis), a mixed-effects model for repeated measures (MMRM) was conducted, which adjusts for repeated assessments per patient over time as well as baseline PRO score and covariates [23]. The MMRM analysis population included patients with change-from-baseline scores; baseline PRO score, stratification factor (cytogenetic response), treatment arm, study visit, and interaction of treatment arm and study visit were included in the models as fixed effects; patient was included as a repeated effect. An unstructured covariance matrix was used as recommended for repeated-measures models [23]. The overall mean change in each arm indicates overall improvement or worsening, and the difference between the arms can be used to quantify the effect of treatment with asciminib compared to bosutinib on PRO score.

The consistency of the longitudinal analysis results (changes over time and difference between treatments) was explored for the following pre-specified subgroups of patients based on key demographic variables and TKI treatment history: sex, race, age, reason for prior TKI discontinuation, and number of prior TKI lines of therapy.

## Results

### Patient characteristics

Of the 233 randomized patients in ASCEMBL, 157 were randomized to receive asciminib and 76 to bosutinib. Overall, the median age at baseline was 52 years (range 19–83) and approximately half (*n* = 120, 52%) were female. A total of 149 (64%) and 81 (35%) patients had discontinued their prior TKI due to lack of efficacy and tolerability, respectively. Full demographic and clinical information for all randomized patients has been presented previously [11].

### PRO completion rate

PRO data were collected for the majority of randomized patients at baseline (96% asciminib and 92% bosutinib). By week 48, there is a larger proportion of patients expected at the clinical visits in the asciminib arm (105 [67% of randomized]) compared to 22 patients (29% of those randomized) in the bosutinib arm due to greater treatment discontinuation in the bosutinib arm. The proportion of the expected patients at each visit completing the MDASI-CML was ≥80% at all visits in each treatment arm (Table 1).

**Table 1:** Completion rate by clinical visit (week) (MDASI-CML).

Timepoint	Completion rate (out of expected population^a^)	
	Asciminib (*N* = 157)	Bosutinib (*N* = 76)
Screening/Baseline	151/157 (96.2%)	70/76 (92.1%)
Week 4	140/152 (92.1%)	65/72 (90.3%)
Week 8	132/149 (88.6%)	61/68 (89.7%)
Week 12	126/143 (88.1%)	55/65 (84.6%)
Week 16	118/140 (84.3%)	51/60 (85.0%)
Week 24	108/130 (83.1%)	41/50 (82.0%)
Week 36	87/109 (79.8%)	24/30 (80.0%)
Week 48	89/105 (84.8%)	21/22 (95.5%)

^a^Expected population includes patients who are pre-progression, alive, and have not withdrawn from the study or switched treatment.

PRO completion rates by visit for the EQ-5D-5L and PGIC-CML questionnaires were similar to the MDASI-CML. For the WPAI-CML, 150/157 (95.5%) and 69/76 (90.8%) patients in the asciminib and bosutinib arms, respectively, reported their current employment status. Of those, 46% (*n* = 69/150) and 36% (*n* = 25/69) in the asciminib and bosutinib treatment arms, respectively, reported currently working when asked at baseline. Only 27 employed patients in the asciminib arm and 8 employed patients in the bosutinib arm completed the WPAI-CML at week 48.

### Patients’ symptoms and HRQOL at start of study

**Fig. 2:**
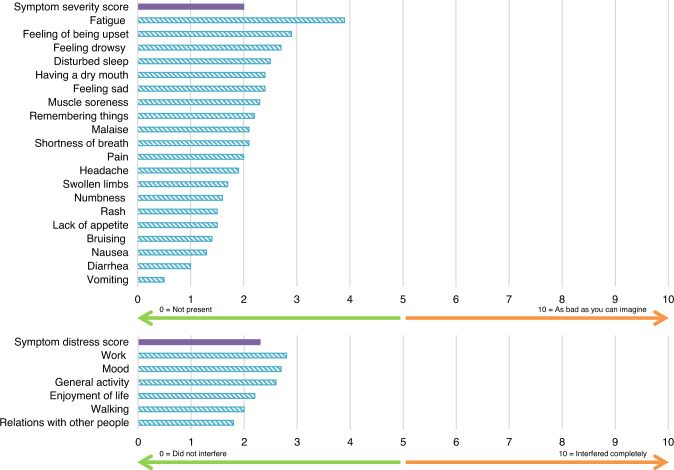
MDASI-CML Specific Symptom and Interference Item Scores at baseline (all patients, both treatment arms, *N* = 221 patients with baseline assessment^*^). ^*^A total of 12 patients were missing data at baseline and not included here.

The mean MDASI-CML symptom severity score at baseline was 2.0 points, indicating that overall symptoms reported by patients before the start of treatment were of a relatively low severity. Fatigue was the most severe symptom reported at baseline with a mean score of 3.9 points. Patients reported that CML interfered with their daily life slightly; the mean symptom distress score was 2.3 points, and work, mood, and general activity were noted to have the most interference out of the six interference items (Fig. 2). There were no meaningful differences in MDASI-CML baseline scores between randomized treatment arms (Supplementary Table S1); therefore, combined baseline data are presented.

Considering general HRQOL as measured by the EQ-5D-5L VAS, patients in both treatment arms combined started the study with a mean VAS score of 72.2 points. Baseline EQ-5D-5L VAS scores were similar between treatment arms at baseline (Supplementary Table S1).

Patients’ general activity impairment level on the WPAI-CML at baseline was 27.1%. Overall work impairment was 23.4% at baseline in those patients who indicated currently working; impairment while working was 20.3%, and work time missed was 16.1%, generally in line with the patients’ reported symptom distress (interference) as assessed using the MDASI-CML questionnaire (Supplementary Table S1).

### Change from baseline over time in PRO scores

#### Change in symptom severity and symptom distress (MDASI-CML)

The change from baseline over time using each of the item, symptom severity, and symptom distress scores (assessed using the MDASI-CML questionnaire up to week 48) in each treatment arm and the difference between the treatment arms in the overall mean PRO scores (least squares [LS] mean) were analyzed.

**Fig. 3:**
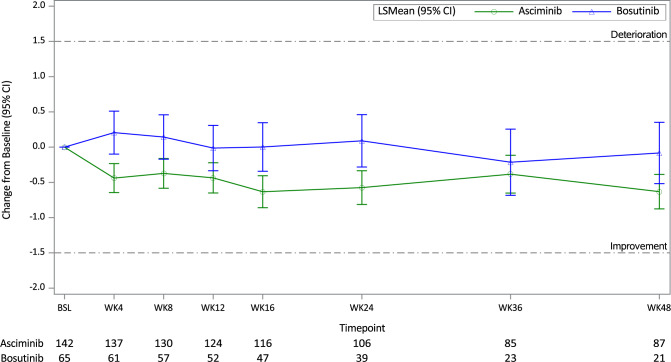
Change from Baseline in MDASI-CML Symptom Severity Score (MMRM analysis). BSL = baseline, CI = confidence interval, LS mean=least squares mean, WK = week. Score ranges from 0 to 10, with a higher score indicating greater symptom severity/interference. Number of patients remaining on treatment at each timepoint shown for asciminib and bosutinib. Negative change shows improvement; positive change indicates deterioration.

Figure 3 presents the change from baseline in the MDASI-CML symptom severity score over time for each of the treatment arms, with the number of patients remaining on treatment stated below the figure. Figure 3 illustrates that the symptom severity score in the asciminib arm showed a trend to decrease after starting asciminib treatment and that symptoms remained stable throughout asciminib treatment (a decrease in symptoms relates to improvement). The symptom severity score in the bosutinib arm stayed close to baseline over the 48 weeks. It is noted, however, that the magnitude of mean changes over time in each of the treatment arms was small, and changes did not reach a clinically meaningful threshold (using 1.5 points as a guide threshold of interpretation).

Patients in the asciminib arm maintained or showed a decreasing trend in severity in all individual MDASI-CML symptom items (demonstrating decreasing severity in 15 out of 20 symptoms); the most notable decreases in symptom severity reported in the overall timeframe were in the symptoms of fatigue (which was the highest at baseline) and having a dry mouth, although these decreases in scores were not clinically meaningful (Supplementary Table S2). Improvements in these symptoms were observed soon after treatment initiation, and the trend for improvement was maintained throughout the 48 weeks (with a reduction in severity of at least 0.9 points at all post-baseline timepoints for fatigue).

In contrast, patients’ scores in the bosutinib arm remained stable for the majority of symptoms, with increases in severity observed for the symptoms of diarrhea, nausea, and vomiting. For diarrhea, an increase of severity from baseline in bosutinib-treated patients of at least 1 point at all timepoints was reported, with the greatest increase in severity observed as early as week 4 (2.6 points more severe compared to baseline). The overall mean increase in diarrhea severity score was 1.5 points, suggesting that it may be a clinically meaningful worsening (based on the MID threshold of 1.5 points) (overall change from baseline data presented in Supplementary Table S2).

**Fig. 4:**
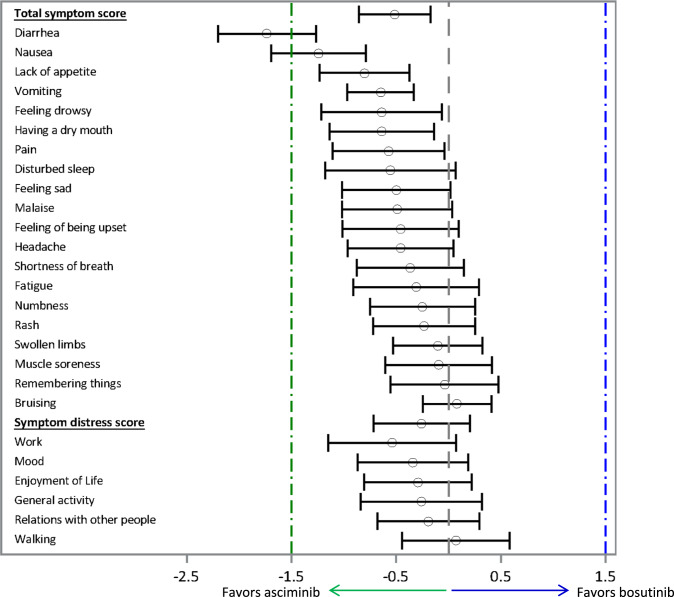
MDASI-CML symptom and interference items: the difference between treatment arms in change from baseline scores. CI = confidence interval, LS Mean=least squares mean. LS mean (95% CI) for difference in change from baseline scores between treatment arms. Dashed lines = clinically meaningful differences (green in favor of asciminib; blue in favor of bosutinib).

The difference between treatment arms in the MDASI-CML mean symptom and interference scores (per MMRM analysis of 48-week data) are shown in Fig. 4.

The difference in change from baseline scores between treatments shows a greater decrease in the symptom severity score, symptom distress score, and in almost all individual symptom and interference items for patients randomized to asciminib treatment compared to bosutinib treatment (LS mean difference <0 favors patients treated with asciminib), although most differences in scores did not reach a clinically meaningful difference (based on MID of 1.5 points). The greatest reported mean (95% CI) differences between treatment arms in favor of asciminib were in the symptom items of diarrhea (−1.7 [−2.2, −1.3]), nausea (−1.2 [−1.7, −0.8]), lack of appetite (−0.8 [−1.2, −0.4]), vomiting (−0.6 [−1.0, −0.3]), feeling drowsy (−0.6 [−1.2, −0.1]), dry mouth (−0.6 [−1.1, −0.1]), and pain (−0.6 [−1.1, −0.0]). Among the symptom items, diarrhea had the greatest difference in score between treatment arms of −1.7 points (indicating a clinically meaningful difference based on the MID of 1.5 points), which was driven by the increase in diarrhea severity reported by patients in the bosutinib treatment arm (Supplementary Table S2).

#### Change in general HRQOL and patients’ perception of change

Overall HRQOL as assessed by the EQ-5D-5L VAS remained similar to baseline in both arms during treatment. As observed in the analysis of MDASI-CML symptom and interference scores, there was a trend of improvement from baseline in HRQOL reported in the asciminib arm (4.8 points on the 0–100 VAS [95% CI 2.4, 7.2]), although it did not reach the published clinically meaningful threshold (MID of 7 points). In addition, there was no clinically meaningful difference in changes from baseline between treatment arms (treatment difference 3.9 [−0.5, 8.2]) (Supplementary Table S2).

The PGIC-CML is a single question assessing patient self-report of change in CML symptom severity since the start of treatment. Considering all patients randomized on each arm (*N* = 157 asciminib and *N* = 76 bosutinib), by week 48, 47% of patients on the asciminib arm reported that their CML symptoms had improved since starting treatment versus 20% in the bosutinib arm. Of note, very few patients on either treatment arm (*n* ≤ 6, <4%) reported any worsening of their CML symptoms at any timepoint (Supplementary Fig. S1).

#### Change in work productivity and activity impairment

Patients in the asciminib arm reported a reduction in their activity impairment (6.5% reduction in impairment, compared to a 1.0% reduction in patients in the bosutinib arm). The analysis of impairment at work was limited due to the low proportion of patients who reported they were currently working. Trends similar to the results of the MDASI-CML interference score analysis were noted in the changes in percent work time missed, impairment while working, and overall work impairment, with trends of differences between treatments favoring asciminib, although the magnitudes of those mean changes were not considered clinically meaningful based on a 15% threshold (Supplementary Table S2).

#### Changes from baseline across subgroups

Changes from baseline in CML symptoms, interference in life, and HRQOL observed across specific subgroups of patients (based on sex, race, age, reason for prior TKI discontinuation, and number of prior TKI lines of therapy) were largely consistent with the results observed for the overall population in ASCEMBL across all PRO questionnaires. A trend for improvement in symptom severity from baseline in favor of asciminib was observed for CML-CP patients, regardless of whether prior TKI therapy was discontinued due to TKI failure or intolerance, though the trend toward improvement in HRQOL from baseline in favor of asciminib was greater for those with prior TKI failure (data not shown).

## Discussion

PRO assessments were pre-specified exploratory endpoints in ASCEMBL and, as such, were not powered to demonstrate significance; therefore, interpreting changes in PRO scores over time as well as differences in mean scores between treatment arms requires consideration of clinically meaningful thresholds. The MDASI User Guide describes that several MIDs have been reported in the literature; the user guide tentatively proposes a guide for MIDs to range from 0.98 to 1.21 [ref. [16]]. However, there are no published data for the MDASI-CML items. In the absence of published MID estimates, calculation of meaningful change thresholds from baseline data using half standard deviation of the baseline value is sometimes applied [24]. Retrospective review of baseline data in ASCEMBL for MDASI-CML items indicated that half baseline standard deviation values ranged from 0.8 to 1.6. Certain HTA agencies such as Institut für Qualität und Wirtschaftlichkeit im Gesundheitswesen (IQWiG, Berlin, Germany) propose a generic rule to use 15% of the total score range for interpretation [21]. Therefore, for the purposes of this analysis, the threshold of 1.5 points or 15% of the total score range that was used as a guide for interpretation seems reasonable and conservative.

In ASCEMBL, mean baseline PRO scores indicated a low impact of CML on symptoms and HRQOL, with mean MDASI-CML baseline scores of around 2 on a scale ranging from 0 to 10 (where higher scores indicate greater severity/interference). Fatigue was the most severe symptom reported at baseline; impact on work, mood, and general activity contributed most to overall symptom distress. At baseline, general HRQOL as assessed by the EQ-5D-5L VAS in this CML-CP population (72.2) was slightly better than a cancer population normative value (68 points for the United States) [22], but worse than the United States general population norm (79.3) [ref. [25]].

During 48 weeks of treatment, symptoms of CML decreased in severity after treatment with asciminib, particularly for fatigue, which started to improve soon after treatment initiation. In contrast, patients treated with bosutinib reported an increase in severity of diarrhea, nausea, and vomiting, with the increase in diarrhea severity observed as early as week 4 and reaching a clinically meaningful threshold overall. These findings are consistent with the 24-week analysis of HRQOL from ASCEMBL [15].

Although LS mean changes from baseline were small and improvements did not meet the clinically meaningful threshold for most items, there is a trend for more improvement in all MDASI-CML symptom and interference items and EQ-5D-5L VAS after treatment with asciminib relative to bosutinib. A larger proportion of patients treated with asciminib compared to bosutinib reported improvement on the PGIC-CML after 48 weeks of treatment. This trend for more improvement in PRO scores for patients treated with asciminib compared to bosutinib is consistent with the 48-week clinical data previously presented, which support better safety and tolerability of asciminib, likely due to its specific mechanism of action. In particular, rates of adverse events leading to discontinuation at week 48 were < 1/3 of those in patients treated with asciminib (7.1%) compared to bosutinib (25.0%) [ref. [14]]. Adverse events leading to dose reduction and interruption, respectively, occurred in fewer patients receiving asciminib compared to bosutinib (23.1% versus 44.7% and 40.4% versus 60.5%, respectively) after 48 weeks [14], further supporting the HRQOL trends observed.

By week 48, the number of patients in the bosutinib arm who were ongoing and expected to complete PRO assessments had dropped to 22, and this small sample size limits our interpretation of treatment comparison data between the two arms. Furthermore, the small proportion of patients who were employed at baseline (asciminib: 46%; bosutinib: 36%) limits our interpretation of the impact of treatment on work productivity, with only 27 and 8 employed patients on asciminib and bosutinib, respectively, responding to WPAI items at week 48. As a result, no clear differences between treatment arms were observed in impact on work as assessed by the WPAI-CML.

In a targeted literature review conducted in 2022, a survey of CML patients on TKIs who completed the MDASI-CML reported that the most commonly prevailing symptom was fatigue (reported by 72.4% of patients) [26], which is the symptom that showed the greatest improvement from baseline on the MDASI-CML following treatment with asciminib in ASCEMBL. Only a single study reporting HRQOL assessment in a third-line setting in CML was identified in the literature, in which the symptom of fatigue was identified as the most important independent predictor of health utility; however, the use of different PRO assessments in the study makes the comparison of findings challenging [27]. Finally, comparisons of asciminib with other practical treatment options for patients with resistant/intolerant CML-CP, such as ponatinib or allogeneic hematopoietic stem cell transplantation, should be evaluated in future studies to better understand the treatment benefit of asciminib in the real-life setting [28]. The inclusion of PRO assessments in routine clinical practice is also important for understanding patient care and treatment burden in the real world [7].

## Conclusion

Patients with resistant/intolerant CML-CP treated with asciminib in ASCEMBL showed a trend for improvement in CML disease- and treatment-related symptoms and HRQOL compared with baseline and relative to bosutinib within the first 48 weeks of treatment. Asciminib-treated patients did not report worsening of treatment-related symptoms, and asciminib treatment did not interfere with patients’ general life activities. These findings are consistent with 48-week clinical data, which support better safety and tolerability in the asciminib treatment arm [14]. No observed deterioration in CML-specific symptoms, HRQOL, or work productivity aligns with the manageable and well-tolerated adverse event profile of asciminib, further supporting the clinical value and risk benefit evaluation of asciminib for the treatment of patients with CML-CP previously treated with ≥2 TKIs.

## Supplementary information

Supplemental information

## Data Availability

Novartis is committed to sharing access to patient-level data and supporting clinical documents from eligible clinical trials with qualified external researchers upon request. These requests are reviewed and approved by an independent review panel based on scientific merit. All data provided are anonymized to respect the privacy of patients who have participated in the trial consistent with applicable laws and regulations. The datasets generated during and/or analyzed during the current trial are available according to the criteria and process described on www.clinicalstudydatarequest.com.
