# Supplementary material for: Health-related quality of life of patients with resistant/intolerant chronic phase chronic myeloid leukemia treated with asciminib or bosutinib in the phase 3 ASCEMBL trial
Source: Leukemia. 2023 Apr 14;37(5):1060–7. doi: 10.1038/s41375-023-01888-y (PMC10169655; doi:10.1038/s41375-023-01888-y)
Supplement: Supplementary file 1 — Supplemental information [file 41375_2023_1888_MOESM1_ESM.pdf]

Supplementary Table S1. Mean (standard deviation) questionnaire scores at baseline

Questionnaire score	Overall (treatments combined) (N=233)	Asciminib (N=157)	Bosutinib (N=76)
**MDASI-CML Symptom Severity Score***	**2.0 (1.7)**	**2.0 (1.8)**	**1.9 (1.7)**
Pain	2.0 (2.7)	2.0 (2.6)	2.1 (2.9)
Fatigue	3.9 (3.1)	4.0 (3.1)	3.8 (3.1)
Nausea	1.3 (2.4)	1.3 (2.4)	1.2 (2.3)
Disturbed sleep	2.5 (2.9)	2.7 (3.0)	1.9 (2.6)
Feeling of being upset	2.9 (3.2)	2.8 (3.1)	3.1 (3.3)
Shortness of breath	2.1 (2.7)	2.1 (2.6)	2.0 (2.7)
Remembering things	2.2 (2.6)	2.2 (2.6)	2.1 (2.6)
Lack of appetite	1.5 (2.6)	1.7 (2.7)	1.2 (2.2)
Feeling drowsy	2.7 (2.9)	2.8 (3.0)	2.3 (2.7)
Having a dry mouth	2.4 (2.9)	2.7 (3.0)	1.9 (2.6)
Feeling sad	2.4 (3.0)	2.3 (3.0)	2.6 (3.1)
Vomiting	0.5 (1.6)	0.5 (1.5)	0.6 (1.9)
Numbness	1.6 (2.6)	1.5 (2.4)	1.8 (2.9)
Diarrhea	1.0 (2.1)	1.0 (2.1)	0.8 (2.0)
Swollen limbs	1.7 (2.6)	1.8 (2.6)	1.4 (2.5)
Rash	1.5 (2.4)	1.6 (2.5)	1.1 (2.0)
Muscle soreness	2.3 (2.9)	2.3 (2.9)	2.2 (2.9)
Bruising	1.4 (2.3)	1.2 (2.2)	1.7 (2.5)
Malaise	2.1 (2.8)	2.0 (2.7)	2.4 (3.2)
Headache	1.9 (2.9)	1.9 (2.9)	1.9 (3.0)
**MDASI-CML Symptom Distress Score***	**2.3 (2.6)**	**2.2 (2.6)**	**2.5 (2.7)**
General activity	2.6 (3.0)	2.6 (3.0)	2.6 (3.1)
Mood	2.7 (3.0)	2.6 (3.0)	3.0 (3.0)
Work	2.8 (3.2)	2.8 (3.2)	2.7 (3.3)
Relations with other people	1.8 (2.7)	1.6 (2.7)	2.0 (2.9)
Walking	2.0 (2.8)	1.8 (2.7)	2.3 (3.1)
Enjoyment of life	2.2 (3.1)	2.1 (3.0)	2.5 (3.2)
**EQ-5D-5L VAS†**	**72.2 (20.8)**	**71.3 (21.7)**	**74.2 (18.8)**
**WPAI-CML‡**			
Work time missed due to CML	16.1% (31.4)	20.3% (34.9)	3.1% (8.1)
Impairment while working due to CML	20.3% (29.9)	22.6% (31.1)	13.8% (26.2)
Overall work impairment due to CML	23.4% (32.8)	26.3% (34.4)	15.6% (27.2)
Activity impairment due to problem	27.1% (29.5)	28.3% (30.3)	24.5% (27.6)

CML=chronic myeloid leukemia; EQ-5D-5L=five-level EQ-5D; MDASI-CML=M. D. Anderson Symptom Inventory – CML; VAS=visual analogue scale

*Score ranges from 0 to 10, with a higher score indicating greater symptom severity/interference.

†Score ranges from 0 to 100, with a higher score indicating a better health state.

‡Score ranges from 0% to 100%, with a higher score indicating greater impairment.

Supplementary Table S2. Overall change from Baseline in PRO scores up to Week 48: CML symptoms and interference (MDASI-CML), general HRQOL (EQ-5D-5L VAS), and work impairment (WPAI-CML)

PRO Score	Average Change from baseline (LS Mean [95% CI])		Difference between treatments in average change from baseline (LS Mean Difference [95% CI])
	Asciminib (N=157)	Bosutinib (N=76)	
**MDASI-CML**	**Negative change indicates improvement (i.e., fewer symptoms or interference)**		**Negative difference favors asciminib**
Symptom Severity Score	-0.5 (-0.7, -0.3)	0.0 (-0.3, 0.3)	-0.5 (-0.9, -0.2)
Pain	-0.4 (-0.7, -0.1)	0.2 (-0.3, 0.7)	-0.6 (-1.1, -0.0)
Fatigue	-1.1 (-1.5, -0.8)	-0.8 (-1.3, -0.3)	-0.3 (-0.9, 0.3)
Nausea	-0.5 (-0.8, -0.3)	0.7 (0.3, 1.1)	-1.2 (-1.7, -0.8)
Disturbed sleep	-0.3 (-0.7, 0.0)	0.2 (-0.3, 0.8)	-0.6 (-1.2, 0.1)
Feeling of being upset	-0.9 (-1.2, -0.6)	-0.4 (-0.9, 0.0)	-0.5 (-1.0, 0.1)
Shortness of breath	-0.5 (-0.8, -0.2)	-0.1 (-0.6, 0.3)	-0.4 (-0.9, 0.1)
Remembering things	-0.2 (-0.5, 0.1)	-0.2 (-0.6, 0.3)	-0.0 (-0.6, 0.5)
Lack of appetite	-0.4 (-0.7, -0.2)	0.4 (-0.0, 0.7)	-0.8 (-1.2, -0.4)
Feeling drowsy	-0.4 (-0.8, -0.1)	0.2 (-0.3, 0.7)	-0.6 (-1.2, -0.1)
Having a dry mouth	-0.9 (-1.2, -0.6)	-0.3 (-0.7, 0.1)	-0.6 (-1.1, -0.1)
Feeling sad	-0.8 (-1.1, -0.5)	-0.3 (-0.7, 0.2)	-0.5 (-1.0, 0.0)
Vomiting	-0.2 (-0.4, -0.0)	0.4 (0.2, 0.7)	-0.6 (-1.0, -0.3)
Numbness	-0.2 (-0.5, 0.1)	0.1 (-0.4, 0.5)	-0.2 (-0.7, 0.3)
Diarrhea	-0.2 (-0.5, 0.0)	**1.5 (1.1, 1.9)***	**-1.7 (-2.2, -1.3)***
Swollen limbs	-0.4 (-0.7, -0.2)	-0.3 (-0.7, 0.0)	-0.1 (-0.5, 0.3)
Rash	-0.0 (-0.3, 0.3)	0.2 (-0.2, 0.6)	-0.2 (-0.7, 0.3)
Muscle soreness	-0.6 (-0.9, -0.3)	-0.5 (-0.9, -0.1)	-0.1 (-0.6, 0.4)
Bruising	-0.4 (-0.6, -0.3)	-0.5 (-0.8, -0.2)	0.1 (-0.2, 0.4)
Malaise	-0.4 (-0.7, -0.1)	0.1 (-0.4, 0.5)	-0.5 (-1.0, 0.0)
Headache	-0.5 (-0.8, -0.2)	-0.0 (-0.5, 0.4)	-0.5 (-1.0, 0.0)
Symptom Distress Score	-0.5 (-0.7, -0.2)	-0.2 (-0.6, 0.2)	-0.3 (-0.7, 0.2)
General activity	-0.6 (-0.9, -0.3)	-0.4 (-0.9, 0.1)	-0.3 (-0.8, 0.3)
Mood	-0.7 (-1.0, -0.4)	-0.3 (-0.8, 0.1)	-0.3 (-0.9, 0.2)
Work	-0.6 (-0.9, -0.2)	-0.0 (-0.6, 0.5)	-0.5 (-1.2, 0.1)
Relations with other people	-0.4 (-0.6, -0.1)	-0.2 (-0.6, 0.3)	-0.2 (-0.7, 0.3)
Walking	-0.2 (-0.5, 0.0)	-0.3 (-0.7, 0.1)	0.1 (-0.4, 0.6)
Enjoyment of life	-0.3 (-0.6, -0.0)	-0.0 (-0.5, 0.4)	-0.3 (-0.8, 0.2)
**EQ-5D-5L**	**Positive change indicates improvement (i.e., better HRQOL)**		**Positive difference favors asciminib**
VAS	4.8 (2.4, 7.2)	0.9 (-2.8, 4.6)	3.9 (-0.5, 8.2)

PRO Score	Average Change from baseline (LS Mean [95% CI])		Difference between treatments in average change from baseline (LS Mean Difference [95% CI])
	Asciminib (N=157)	Bosutinib (N=76)	
**WPAI-CML**	**Negative change indicates improvement (i.e., less impairment)**		**Negative difference favors asciminib**
Activity impairment	-6.5% (-9.6, -3.5)	-1.0% (-5.9, 3.8)	-5.5% (-11.1, 0.1)
Work time missed[†]	-0.1% (-5.0, 4.8)	-5.0% (-14.2, 4.1)	4.9% (-5.4, 15.3)
Impairment while working[†]	-6.8% (-10.9, -2.6)	-3.4% (-10.8, 4.0)	-3.4% (-11.8, 5.0)
Overall work impairment[†]	-4.4% (-9.8, 0.9)	-2.3% (-11.8, 7.2)	-2.1% (-12.9, 8.7)

[*]Clinically meaningful changes/differences (based on following thresholds: MDASI-CML=1.5; EQ-5D-5L VAS=7; WPAI-CML=15%)

[†]Items only completed by patients who were working (asciminib n=69 at baseline; bosutinib n=25 at baseline)

Supplementary Figure S1. PGIC-CML Item Response by Timepoint for Asciminib (N=157) and Bosutinib (N=76)

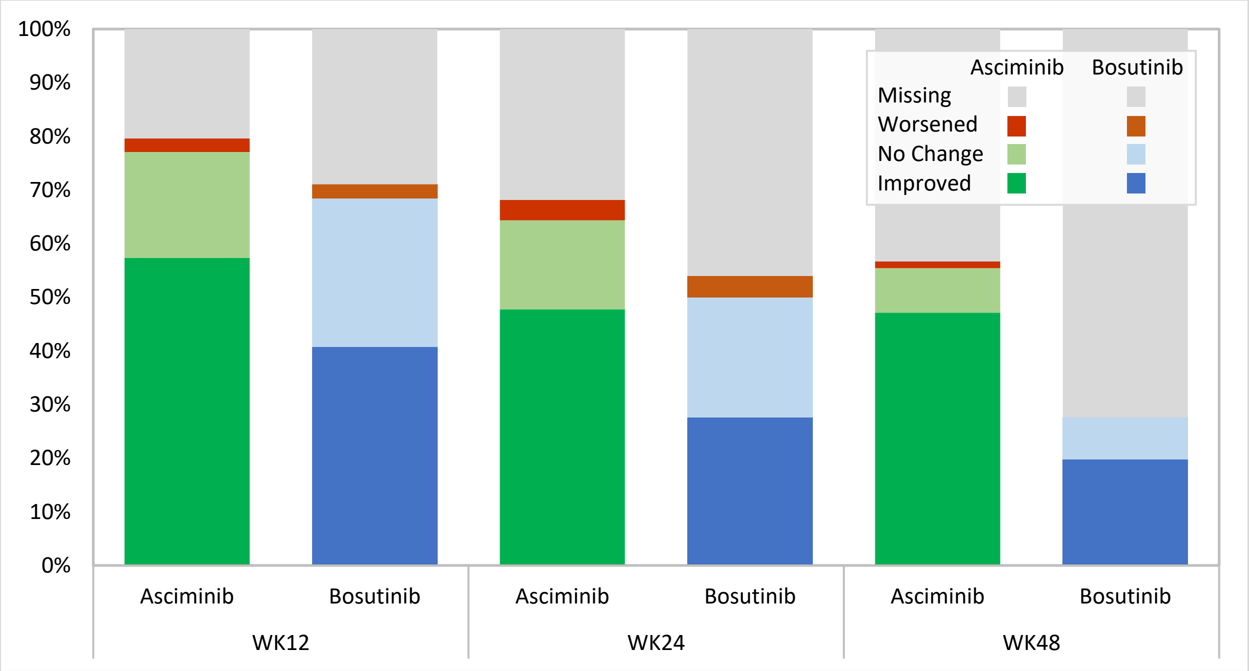

Response groupings based on a seven-point scale from 1 (very much improved) to 7 (very much worse). Improved: responses 1, 2, or 3; Stable: response 4; Worsened: responses 5, 6, or 7
